# Widening participation in cognitive neuroscience: a mixed-methods study of age-related motivators, barriers and attitudes towards imaging methods

**DOI:** 10.1186/s12874-026-02911-3

**Published:** 2026-06-19

**Authors:** Ksenia Kotiusheva, Laurel Hilliker, Tracy Ibbotson, Satu Baylan, Maria Gardani, Gemma Learmonth

**Affiliations:** 1 School of Psychology & Neuroscience, University of Glasgow, Glasgow, UK; 2 School of Education, University of Glasgow, Glasgow, UK; 3 School of Medicine, Veterinary & Life Sciences, University of Glasgow, Glasgow, UK; 4 Regional Neuropsychology Services, Queen Elizabeth University Hospital, Glasgow, UK; 5 School of Health in Social Science, University of Edinburgh, Edinburgh, UK; 6 School of Psychology, University of Stirling, Stirling, UK

**Keywords:** Neuroimaging, Altruism, Motivators, Barriers, Recruitment

## Abstract

**Background:**

Cognitive neuroscience research often relies on convenience sampling of participants, which can result in biased sample demographics and an under-representation of older adults. There is a need to identify more effective routes to widen participation among older adults and to explore age-related differences in the motivators and barriers to research involvement.

**Methods:**

This mixed methods study combined qualitative data from two focus groups, conducted with *N* = 11 healthy older adults aged 55–73, and an online questionnaire completed by *N* = 336 adults aged 18–88.

**Results:**

Analysis of the focus group discussions identified 3 main themes that were most important to older adults: a) The importance of receiving transparent information about the aims, procedures and safety of the study, b) Distinguishing between medical and non-medical research, and c) Contributing to the “collective good”. The questionnaire echoed that altruism, and the prospect of scientific discovery, are increasingly important motivators with advancing age, whereas financial incentives become less important. Older adults have more free time to participate, are less deterred by the prospect of pain, and express more trust in researchers than younger people. Attitudes towards different imaging methods (MRI, EEG, NIBS and Eye tracking) varied, with fewest negative emotions for eye-tracking and most for non-invasive brain stimulation, but positive attitudes generally increased and negative attitudes reduced with age.

**Conclusion:**

These findings can inform age-tailored recruitment strategies to improve diversity in neuroimaging research. Improving communication, addressing practical barriers, and framing studies in a meaningful context may help increase participation among groups who are traditionally underrepresented in neuroimaging research.

**Supplementary Information:**

The online version contains supplementary material available at https://doi.org/10.1186/s12874-026-02911-3.

## Introduction

Our cognitive abilities are susceptible to change as we get older. Although performance is often maintained in some cognitive domains (e.g., verbal reasoning), or may even improve with advancing age (e.g., semantic knowledge), healthy older adults become generally less proficient compared to younger people across a range of functions (e.g., processing speed, spatial visualisation and memory) [36]. These age-related changes have been extensively quantified using behavioural assessments, and more recently, neuroimaging methods have also provided important insights into the neurophysiological mechanisms underlying the aging process.

Given their distinct spatial and temporal resolutions, different neuroimaging modalities are often integrated to more accurately characterise the neural substrates and temporal dynamics of age-related cognitive decline. For example, functional magnetic resonance imaging (fMRI) has identified spatially distributed patterns of cerebral blood flow in older adults that are typically more lateralised to one cerebral hemisphere in young adults. This shift has been interpreted as a compensatory mechanism, whereby additional brain regions are recruited to maintain behavioural performance [9]. Electrophysiological techniques such as magnetoencephalography (MEG) and electroencephalography (EEG) have identified age-related changes in brain rhythms, including a slowing of occipital alpha oscillations, which may reflect reduced visual sampling rates in older adults [37, 42, 43]. In addition, recent advances in non-invasive brain stimulation (NIBS) interventions, such as transcranial magnetic stimulation (TMS), transcranial direct current (tDCS), alternating current (tACS), and random noise stimulation (tRNS), have enabled researchers to probe causal relationships between the modulation of specific brain regions or networks and cognitive performance. Although not a neuroimaging method per se, eye-tracking is also widely used in ageing research to assess gaze patterns and pupil dynamics, serving as an indirect index of arousal, attentional focus, and cognitive strategies [12].

Cognitive neuroscience research must be based on participant samples that are representative of the broader population [21]. While many areas of psychology have adopted online testing platforms to improve accessibility and reach, cognitive neuroscience studies typically require participants to attend specialised research facilities in person. Thus, recruitment often relies on convenience sampling from individuals living near academic institutions. This approach frequently results in samples composed predominantly of university students, who differ from the general population in key demographic variables, including educational attainment, socioeconomic status, and ethnic background [11, 16]. Such sampling biases can limit the extent to which study findings generalise beyond the immediate participant pool and result in sub-optimally effective treatments for those who need them most. Although this issue is often highlighted in studies involving young adults, it is equally relevant to research on older populations. Anecdotally, this has been reflected in our own experiences, where our healthy older adult participants are often retired professionals with high levels of education. This pattern of participant selectivity has also been observed in large-scale biomedical studies such as the UK Biobank, which, despite its national scope, overrepresents individuals who self-define as White, are highly educated, and homeowners [5]. Furthermore, those who agreed to undergo brain imaging as part of the Biobank were found to be healthier in terms of mental health, and other cognitive, cardiometabolic, inflammatory and neurological measures, compared to non-imaged participants [26].

Previous studies have investigated the specific motivators and barriers for older people taking part in clinical trials, often within specific clinical populations [17, 18, 24, 45], and have also examined strategies for increasing participation among under-represented groups more broadly [13]. However, the factors that encourage participation in clinical trials may not directly translate to non-medical, academic research, particularly studies without clear or immediate therapeutic benefits. Within clinical research contexts, a variety of motivators have been reported, including curiosity about the study, a desire to learn more about a health condition, and the perceived personal benefits of receiving health-related assessments [1, 41]. A review of 26 systematic reviews by Sheridan et al., [38] described three primary facilitators for adult participation in health-related research: the potential for personal benefit, altruism, and trust in the research team. The reported barriers were highly context-dependent and varied according to the characteristics of the target population, the research setting, and specific elements of the study design.

Within the context of neuroimaging, Wardlaw et al. [44] investigated public and expert opinions regarding the perceived utility and acceptability of neuroimaging. Among respondents from the general public, 90% reported being willing to undergo brain scanning for scientific purposes, rising to 96% for medical purposes, reflecting remarkably high levels of acceptance of brain scanning in this group. However, willingness declined markedly when imaging was proposed for other purposes, with only 36% of participants willing to undergo scanning for criminal investigations and just 9% for insurance reasons. Thus, the aims and context of neuroimaging appears to play an important role in shaping attitudes towards participation. In addition, trust in the research team and the influence of healthcare professionals have been identified as important facilitators of recruitment in health-related studies [38]. Given the growth of the digital technology sector and the widespread adoption of health-tracking devices over the past decade, neuroimaging may be perceived as even more familiar and acceptable than it was at the time of these earlier studies. Despite this, to date, no investigation has been conducted to explore the specific barriers and motivators influencing healthy older adults' participation in cognitive neuroscience research. Similarly, little is known about older adults’ perceptions of commonly used neuroimaging modalities, or about the most effective strategies for recruiting and communicating with this participant demographic.

In this mixed method study, we aimed to understand healthy older adults’ understanding of, and attitudes towards, four commonly used cognitive neuroscience research techniques: magnetic resonance imaging (MRI), electroencephalography (EEG), non-invasive brain stimulation (NIBS) and eye tracking. A secondary objective was to examine whether these views differ between older and younger adults. Specifically, the study addressed three key research questions: 1) What are older adults’ attitudes towards different cognitive neuroscience methods? 2) What are the primary motivators and barriers influencing their willingness to participate in neuroimaging research studies? and 3) What recruitment strategies and approaches in disseminating research outcomes are perceived as the most effective?

## Methods

### Qualitative methods

#### Methodological approach

The focus group arm of this study was approached using the social constructivist paradigm, which views social reality as subjective and shaped via shared understanding through the research process [19].

#### Participant recruitment

Eleven older participants were recruited as part of two focus groups (Table 1 presents pseudonymised demographics). The first focus group involved 6 adults (4 women, mean age = 66.2, range = 55–73 years old) who all lived in the local, inner-city area, and was held in the Advanced Research Centre at the University of Glasgow. The second focus group involved 5 adults (4 women, mean age = 64.8, range = 62–66) from a small, rural town in Scotland, approximately 50 miles from the nearest city and university, and was held in the home of one of the participants. Participants in the first focus group were purposefully recruited via social media, while participants for the second focus group were recruited through personal contacts. Participants were recruited from rural and urban areas (classified using the Scottish Government Urban–Rural Classification) and organised into two distinct focus groups to explore whether location influenced motivators and barriers to participation in neuroimaging research. A heterogeneous sampling approach [35] was used because the researchers aimed to understand views of older adults from both urban and rural areas. Group sizes of five and six participants were selected to allow sufficient time for in-depth discussion and elaboration by each participant, which may be constrained in larger focus groups [22]. Consistent with reflexive thematic analysis, the aim was to prioritise richness and interpretive depth of data over formal saturation thresholds [7]. The only inclusion criterion was that participants self-reported that they were in good general health, with the rationale that all participants were potentially eligible for recruitment into healthy aging research studies. Participants received a £25 book token to compensate for their time.

**Table 1:** Demographics of participants (pseudonymised) in the urban and rural focus groups

Focus Group 1 (Urban)	Age	Focus Group 2 (Rural)	Age
Joanna	56	Emily	64
Anne	55	Shona	66
Peter	73	Eileen	66
Mary	61	Sarah	62
Gary	73	Michael	66
Natalie	70		

#### Focus groups

A set of semi-structured interview questions was co-developed with 6 community-dwelling older adults from the Public and Patient Involvement & Engagement (PPIE) group at the University of Glasgow, to explore older adults’ understanding and attitudes towards cognitive neuroscience research. The PPIE group was organised and led by TI with GL, KK and LH present and actively contributing to the discussion. Both focus groups were conducted by KK. There was no overlap between participants in the PPI group and those in the focus groups.

The introductory questions were broad and open and were aimed at unpacking the general understanding of research, e.g., “*What do you know about research in general?*” and “*Why do people do research?”.* The second section was split into four themes, with each theme focusing on one commonly used method in cognitive neuroscience research (EEG, MRI, non-invasive brain stimulation and eye tracking, respectively). Participants were asked: “*What is your understanding of [imaging method]?*”, “*When people get an [imaging method], what information does this provide?*”. Short explanatory videos were created by author KK (available at https://osf.io/y9fjk/), providing broad explanations of each method, and were shown to the groups in order to promote further discussions. Participants were asked: “*After watching this video, would you like to share your opinions about [imaging method]?” “What are your thoughts about taking part in this type of research?”.* Finally, a concluding set of questions aimed to identify how researchers might recruit older adults into cognitive neuroscience experiments more effectively: *“We need to advertise our experiments more widely. Where might we advertise to reach more older people?”.*

The interview questions were then delivered to the focus groups and were audio recorded using a digital recorder. The participants were provided with the transcribed discussions for editing prior to analysis, but no edits were requested. At the end of the discussions, participants were asked to provide feedback about the planned questionnaire arm of this project (see Quantitative methods).

#### Qualitative data analysis

The discussions were transcribed verbatim by author KK. All identifiable information was removed, and participants were given pseudonyms. A thematic analysis [6] was used to analyse the data using six key steps: 1) familiarisation with the data, 2) generating initial codes, 3) searching for themes, 4) reviewing themes, 5) defining and naming themes, and 6) producing the report. Two authors (KK and LH) separately analysed the transcripts by familiarising themselves with data under a shared understanding of the analytic aims. They then independently generated initial codes and identified potential themes using NVivo*.* Coding was primarily inductive and conducted at the semantic level. Any differences in coding were resolved through discussion among authors KK, LH and GL, and the overarching themes were agreed through mutual discussion. The study broadly followed Reflexive Thematic Analysis Reporting Guidelines (RTARG, [8].

During the research process, specifically when collecting and analysing the data, the researchers maintained their philosophical viewpoint and engaged in constant dialogues which allowed them to critically appraise emerging themes. Additionally, all researchers are below age 55 and have a diverse cultural background with expertise and/or interests in academic research. Some authors have also worked with aging populations in clinical settings. Such diverse and multi-faceted team characteristics stimulated further discussions related to the research questions.

### Quantitative methods

#### Participants

A total of 336 complete datasets were obtained from adults aged 18–88 living in the United Kingdom via the Prolific research platform (Table 2). The sample was stratified across seven age bins to ensure representation across the full age range of interest (age 18–29: *N* = 52, 35 women, 2 non-binary, median age = 25; age 30–39: *N* = 50, 33 women, median age = 36; age 40–49: *N* = 53, 38 women, median age = 44; age 50–59: *N* = 51, 37 women, median age = 54; age 60–69: *N* = 50, 35 women, median age = 65; age 70–79: *N* = 50, 31 women, median age = 72 and age 80–89: *N* = 30, 18 women, median age = 81). Demographic data was collected on gender, ethnicity, education, employment and the degree of urbanisation of their residential location. The sample predominantly self-identified as White (94.4%) and this proportion is higher than estimates for the UK population overall (approximately 80–85% White). Around one third (32.7%) of the sample was educated to bachelor’s level or higher, which is broadly equivalent to the UK population as a whole. Participants were each paid £1.50 to complete the questionnaire, with a median completion time of 11 min. The study was approved by the College of Medicine, Veterinary and Life Sciences ethics committee at the University of Glasgow and all participants provided informed consent prior to starting the questionnaire.

**Table 2:** Demographics of participants in the quantitative study (*N* = 336)

	***N* (%)**
Sex	
Men	107 (31.9)
Women	227 (67.6)
Non-binary	2 (0.6)
Ethnicity	
White	317 (94.4)
South Asian	6 (1.8)
Mixed	5 (1.5)
Black/African/Caribbean	4 (1.2)
East Asian	3 (0.9)
Arab	1 (0.3)
Education	
Bachelor’s degree	110 (32.7)
Vocational qualifications	66 (19.6)
Secondary school	65 (19.4)
Graduate degree	53 (15.8)
Some university but no degree	30 (8.9)
Some secondary school	10 (3.0)
Completed primary school	1 (0.3)
Preferred not to answer	1 (0.3)
Employment	
In employment	194 (57.7)
Retired	106 (31.6)
Unemployed	19 (5.7)
Student	13 (3.9)
Preferred not to answer	4 (1.2)
Residential location	
Inner city	47 (14.0)
Town	107 (31.9)
Suburban	104 (31.0)
Village	54 (16.1)
Rural or countryside	24 (7.1)

#### Questionnaire

The full questionnaire is available at https://osf.io/y9fjk/. The questionnaire was developed in collaboration with the Patient and Public Involvement & Engagement group at the University of Glasgow, and focus group participants from the qualitative arm of the project. After reviewing the draft questionnaire, the participants provided feedback on the key themes to include, as well as on the wording and phrasing of each question, through a discussion with the research team. The questionnaire comprised 4 broad themes which aimed to understand: 1) The participants’ previous involvement in medical or academic research, 2) Their familiarity with MRI, EEG, NIBS and eye tracking imaging methods, 3) Emotions towards undertaking each imaging method as part of their medical care or a research study, and 4) Barriers and motivators towards taking part in cognitive neuroscience research.

#### Quantitative data analysis

Quantitative analyses were conducted to address three aims: 1) to examine emotional responses to different neuroimaging methods across age and study context, 2) to identify facilitators and barriers associated with willingness to participate in research, and 3) to examine preferences for how participants wish to receive information about research opportunities. Separate statistical models were specified for each aim, as described below. All data analysis was performed in RStudio [32].

The raw Likert scale rating scores (where 0 = “I would not feel this way” and 100 = “I would definitely feel this way”) were extracted separately for the eight emotions that were probed (*excited*, *interested*, *grateful, nervous, scared, stressed, worried,* and *indifferent*)*.* These scores were averaged into three simplified emotion groups: Positive emotions (scores averaged for *excited*, *interested* and *grateful*), Negative emotions (scores averaged for *nervous, scared, stressed* and *worried*) and Neutral emotions (*indifferent*). Across each pair of imaging modalities (MRI, EEG, NIBS and Eye tracking) and settings (medical, research), positive emotion items had moderate-to-good internal consistency (Cronbach’s alpha = 0.66—0.83) and negative items had excellent internal consistency (Cronbach’s alpha = 0.95—0.97). The full outputs are available as an additional file [see Additional file 1].

To examine emotional responses across imaging methods, contexts and age, a linear mixed-effects model was conducted using the *lme4* package [4]. Emotion ratings were modelled as the dependent variable, with *Emotion* (positive, neutral, negative), *Imaging method* (MRI, EEG, NIBS, eye tracking), *Study location* (research, medical), and *Age* (entered as a continuous variable) as fixed effects. Participant ID was included as a random intercept to account for repeated measurements and individual differences in baseline responses. *P*-values for fixed effects were calculated using the Satterthwaite approximation for denominator degrees of freedom, as implemented in the *lmerTest* package, and internal calculation limits were increased to ensure accurate computation given the size of the dataset. Tukey-adjusted pairwise post-hoc comparisons for significant interaction effects were conducted using the *emmeans* package [23].

To examine predictors of willingness to participate in research, two generalized linear mixed effects models with a binomial link function were specified. One model included the 7 facilitator items as fixed effects, and the second included the 11 barrier items, along with participant age as a covariate in both models. Random intercepts were specified for participants to account for individual differences in baseline willingness to participate, and for imaging modality to account for variability across MRI, EEG, NIBS and eye tracking studies. Odds ratios and 95% confidence intervals were calculated to assess the likelihood of participation.

Chi-square tests were used to compare the proportions of participants who were willing to take part in each of the four imaging methods in research versus medical contexts. Finally, linear regressions were used to predict participants’ age, based on their binary (yes/no) preferences for how they might want to receive information about studies (*online, GP, social media, TV, newspaper, radio, in a shop* or *approached*). Average marginal effects and predicted probabilities were computed from fitted regression models using the *marginaleffects* package.

## Results

### Qualitative results

Each focus group lasted for 1 h. A total of 67 pages of single-spaced data was produced (27,546 words). Three major themes identified from the focus groups refer to older adults’ views about 1) the need for information about the research, 2) the importance of the distinction between medical and non-medical research, and 3) altruism or contributing to the collective good (Table 3).

**Table 3:** Summary of the 3 main themes and 6 sub-themes identified in the focus groups

	**Main themes**		**Sub-themes**
**1**	The need for information and transparency	**a**	About the aims of the study
		**b**	About the safety of the procedures
		**c**	About what will happen to them during the study
**2**	Importance of the distinction between medical and non-medical research	**a**	Developing medical treatments *vs* understanding brain mechanisms
		**b**	Using research tests as a health check
		**c**	Having a personal connection to the medical condition(s) that are being studied
**3**	Altruism and contributing to the collective good	-	-

#### Theme 1. The need for information and transparency

Participants described several categories regarding the information they would like to receive before participating in experiments. These include information about a) the aims of the study, b) the safety of the procedures, and c) what will happen to them during the study.

- About the aims of the study

Participants in both focus groups highlighted that the key factors in deciding whether or not to take part in research are the aims of the research project, and what their data would be used for. Many questions arose from participants in relation to the data collection and interpretation. Characteristically a participant asked *“Why do you want the data, really?” (Joanna)* and *“What would be the point of the research?” (Anne).*

Participants also wanted to understand the data analysis process as well as how specific equipment generated data. For example, Natalie questioned how the EEG system recorded brain activity and how researchers were able to interpret it: *“How are they interpreted? Brain waves. Like… You, like it's on… When you get your current. Is it a print out or is there a… what is there to see? Uh, what you what you're looking for?”.* As the importance of having information about research processes became evident during the discussion, Joanna captured this theme succinctly*: “So, for me there has to be a motive. If you're saying to me, come and sit in my living room or stick this on your head for fun. Do you see what I mean?”.*

b) About the safety of the procedures

Safety of the procedures was another key topic discussed by the participants as part of the overarching theme. The need for information was expanded by other participants, who mentioned that they wanted to know more about the safety of the procedures. For example, participants expressed their concerns regarding potential side-effects of non-invasive brain stimulation. They stated that they wanted reassurance that it was “*not gonna have any adverse effect on me” (Emily)* and that they would need a lot of reassurance before agreeing to participating in experiments involving this method: *“What if the pulse burns…You know, I would need to know how safe it was because someone sending a zap into my brain and if it could burn that erm connection to my thumb movement, for example. You know, I’d be really scared, you know” (Joanna).*

c) About what will happen to them during the study

Finally, participants discussed the importance of researchers communicating all relevant information with participants transparently and in a timely manner, highlighting this as a crucial step of making an informed choice about participation: *“I think the kind of information sheet that you sent out before this meeting even is helpful because you see it, you've got time to assess it, pass it to my wife and say what do you think if I want to, I can run it by my son or my daughter (Gary).”*

Furthermore, participants talked about the value of the short videos explaining what participation in cognitive neuroscience experiments with different imaging techniques involved. They emphasised that such videos could serve as an effective communication tool between researchers and participants, potentially encouraging older adults to take part by allowing them to see and hear the researcher’s message and decide whether or not to participate based on how engaging or relevant the study appears:

*“I think that the video… because it's so simple takes any fear away, that anyone would have. Because you can look at that and think, well, you know, I'm going to get messy hair, but who doesn't get messy hair (laughs). So, it's uh… and I think even just showing that tiny little chart of, you know, what's been measured again. Makes it quite interesting even though it's simple. But interesting enough to encourage people, I think, to take part” (Anne).*

#### Theme 2. Distinction between medical and non-medical research

Three sub-themes comprised the next major theme of distinction between medical and non-medical research: a) developing medical treatments vs understanding brain mechanisms, b) research tests as a health check, and c) having a personal connection to the medical conditions that are being studied.

- Developing medical treatments vs understanding brain mechanisms

Participants felt it was important to understand whether the research was a theoretical study to learn more about the brain, or whether the aim was to develop a better knowledge of a medical condition or develop a clinical treatment: *“It’s very important to differentiate as to whether there is a medical objective as opposed to a scientific one or both and what they are. So some of the comments… that you know, there's a fear of some of the procedures and what it might (inaudible) medically rather than scientifically, something is very important to make that erm yeah, differentiation if there is one (Peter).*

Additionally, older adults questioned and discussed applications of methods they were less familiar with, for example, participants were curious about the functions of EEG: “*What you need out of it? What? What you're seeing on the screen, you know, the pulses. I’d like somebody to explain what you're actually getting from that” (Michael).* This point is further illustrated by Peter’s enquiry concerning the purpose of EEG: *“Is it only a measurement process you're describing, or can it also be used as a treatment?” (Peter)*.

b) Research tests as a health check

Some participants further expanded the discussion by exploring the idea of taking part in cognitive neuroscience research experiments as a means of obtaining a medical check-up. For example, one participant shared: “*If that was going to be preventative, if you think in the months it took to get me diagnosed, the amount of money that was spent on me going to doctors or hospitals, medication, I mean that must have come to, you know, thousands of pounds, whereas if the MRI had been offered earlier or if it was part of a routine going forward where things can be picked up before they go” (Anne).*

However, opinions were mixed about whether they would like to see the results of their individual tests*,* signifying the importance of offering participants a choice of whether they wish to see their results: “*Ohh yeah. You want to know the end result.” (Michael)* and *“But personally, I wouldn't want to know if I had dementia, I’d quite happily go through my life not knowing” (Joanna)*.

3) Having a personal connection to the medical condition(s) that are being studied

Another sub-theme that emerged in the discussion was participants’ personal connection to the phenomenon being studied, with some suggesting that they would be more inclined to participate if they had personal experience with it: *“My mum had dementia. So, you know I'm interested from that point of view” (Mary).*

#### Theme 3. Altruism and contributing to the collective good

The final and most notable theme focused on participants motivation to support researchers in what they perceived as meaningful and worthwhile work. All participants highlighted that they wanted to be helpful. For example, Gary said: *“See, I would do it because even though it's not telling me anything necessarily or identifying any issues with me, it's giving information to the researchers and possibly eventually clinicians. So, if I can help with that, fine would be my approach”.*

Finally, a quote made by Peter reflects a desire to contribute to the “collective good” resonated with everyone*: “Is it not the collective that's most important?”.*

#### Comparison of the urban and rural focus groups

One key difference identified between the two focus groups concerned access to information about research, particularly neuroimaging research studies. Participants in the rural group largely relied on personal connections, such as family members of their friends. In contrast, participants in the urban group reported greater access to such information through their workplaces or other sources in their immediate environment. This distinction is illustrated in the following quotations:

*Emily (Rural)* *:* *Erm sort of, erm brain research here, just like Eileen, we’re saying it's only cause of Sarah, you know? Erm. I have never heard it. I know it goes on, but I've never heard it roundabout here or the opportunity to take part roundabout here.*

Joanna (Urban): *“And there's a hospital that looks into dementia, and people are allowed to just make an appointment and go and have various test done”*.

### Quantitative results

#### Prior experience of research

A large majority of the 336 respondents had taken part in a research study before (79.2%, *N* = 266). These studies had mostly taken place in a university setting (57.9%, *N* = 152), followed by a hospital or medical setting (30.1%, *N* = 80), and 12.8% (*N* = 34) had taken part in both university and hospital-based research studies. A logistic regression identified that for each additional year of age, the odds of having participated in a previous research study decreased by 2.47% (*β* = −0.025, *p* = 0.0011).

The majority of participants (63.4%, *N* = 213) had not received or undertaken any type of imaging procedure previously, either for medical or research purposes. Of the 165 (49.1%) respondents who had received some form of imaging, more than two thirds (52.1%, *N* = 86) had undergone an MRI scan, 22.4% (*N* = 37) had undertaken an eye tracking procedure, 21.2% (*N* = 35) had received an EEG, and only 4.2% (*N* = 7) of respondents had experienced non-invasive brain stimulation. Of note, almost a third (26.1%, *N* = 43) of our sample who had received imaging had experienced more than one type of imaging method.

Respondents generally had low familiarity with brain imaging methods. More than half (55.7%, *N* = 187) stated that they were not familiar at all, 39.3% (*N* = 132) knew a little whereas 4.8% (*N* = 16) were reasonably familiar and 1 person (0.3%) was extremely familiar with brain imaging methods. Around 50% of respondents aged under 60 years old had no familiarity with brain imaging methods, and this increased to around 70% in those aged over 60 years old.

#### Perceptions of cognitive neuroscience methods

**Fig. 1:**
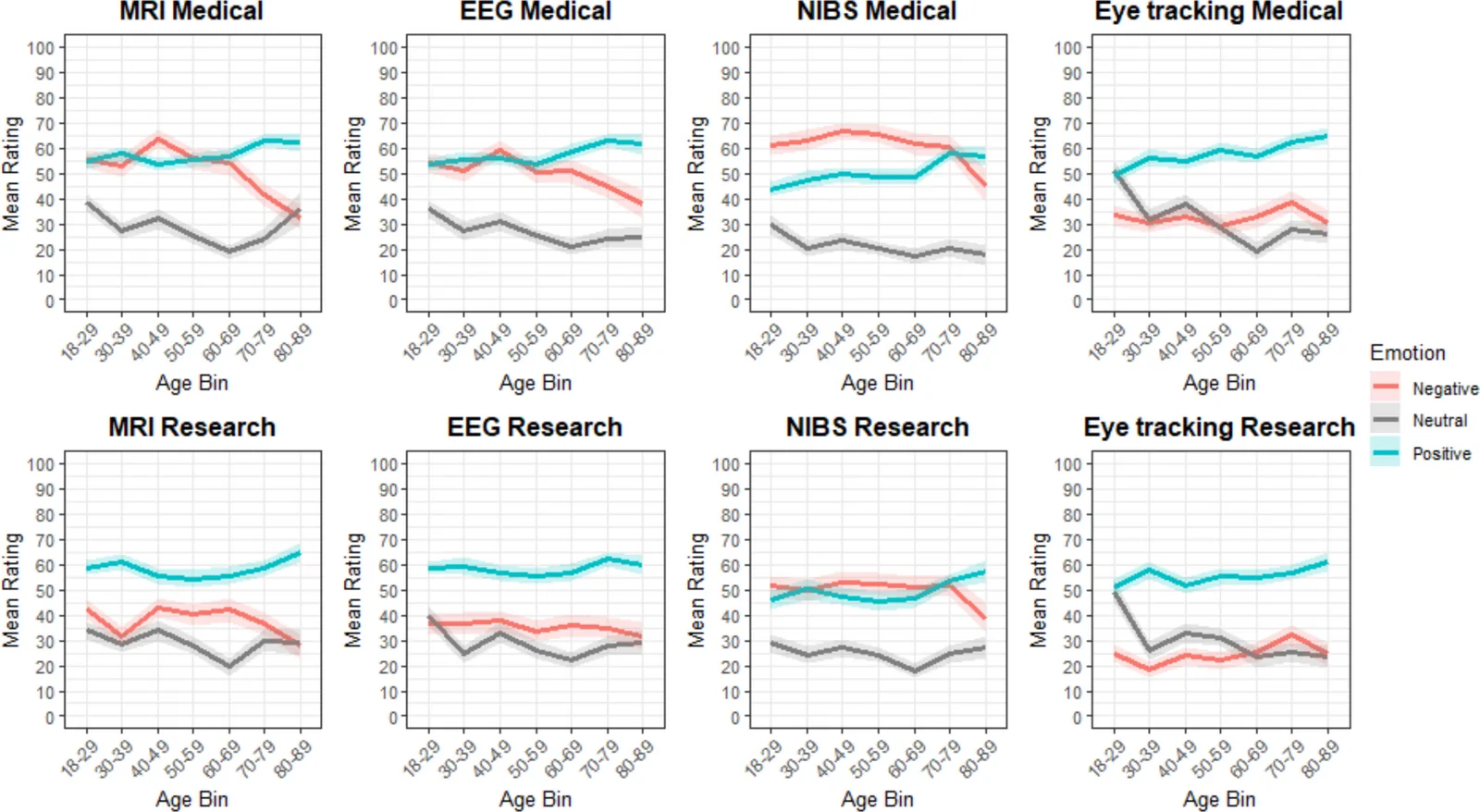
Mean ratings in response to the question “*How do you think you might feel if you were asked to get a [imaging method] as part of [your medical care/a research study]”.* Ratings were averaged for positive emotions (excited, interested and grateful), negative emotions (nervous, scared, stressed and worried) and neutral (indifferent), where 0 = “I would not feel this way” and 100 = “I would definitely feel this way”. Shaded error bars represent ± 1 standard error of the mean. MRI = magnetic resonance imaging, EEG = electroencephalography, NIBS = non-invasive brain stimulation

The median rating for positive, neutral and negative emotions is shown in Fig. 1, across each of the 7 age bins and separately for studies taking place in medical and research settings. There were strong main effects of *emotion* [F(2, 21,122) = 17.88, *p* < 0.0001], *imaging method* [F(3, 21,122) = 7.15, *p* < 0.0001] and *location* [F(1, 21,122) = 14.85, *p* = 0.00012] on rating scores, but no main effect of *age* [F(1, 360) = 3.19, *p* = 0.075].

**Fig. 2:**
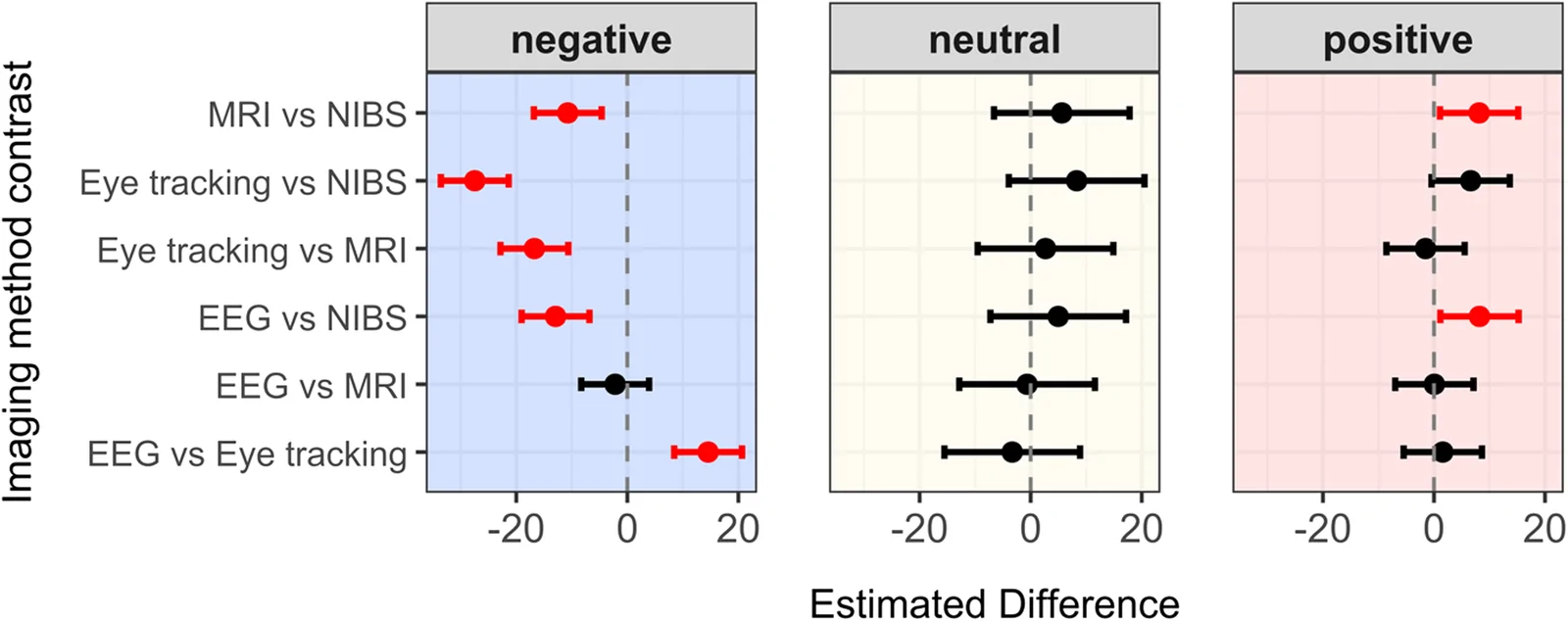
Contrast estimates and 95% confidence intervals for the pairwise comparisons of imaging method ratings, separately for negative, neutral and positive emotions. The comparisons where *p* < 0.05 are highlighted in red and *p* > 0.05 in black. MRI = magnetic resonance imaging, EEG = electroencephalography, NIBS = non-invasive brain stimulation

Since one of our primary goals was to assess whether the four imaging methods evoke different emotional attitudes, our first interaction of interest was *imaging method* x *emotion* [F(6, 21,122) = 43.47, *p* < 0.0001]. Paired comparisons revealed distinct clusters of differences in ratings (Fig. 2). Broadly, non-invasive brain stimulation was rated more negatively compared to all three other imaging methods, and less positively than MRI and EEG. Eye tracking was rated less negatively than both EEG and MRI.

Second, we were interested in whether the location of the study evoked different emotions (*location* x *emotion*: [F(2, 21,122) = 55.95, *p* < 0.0001]. Medical settings evoked higher ratings of negative emotions compared to research settings [t(21,122) = 4.3, *p* < 0.0001].

**Fig. 3:**
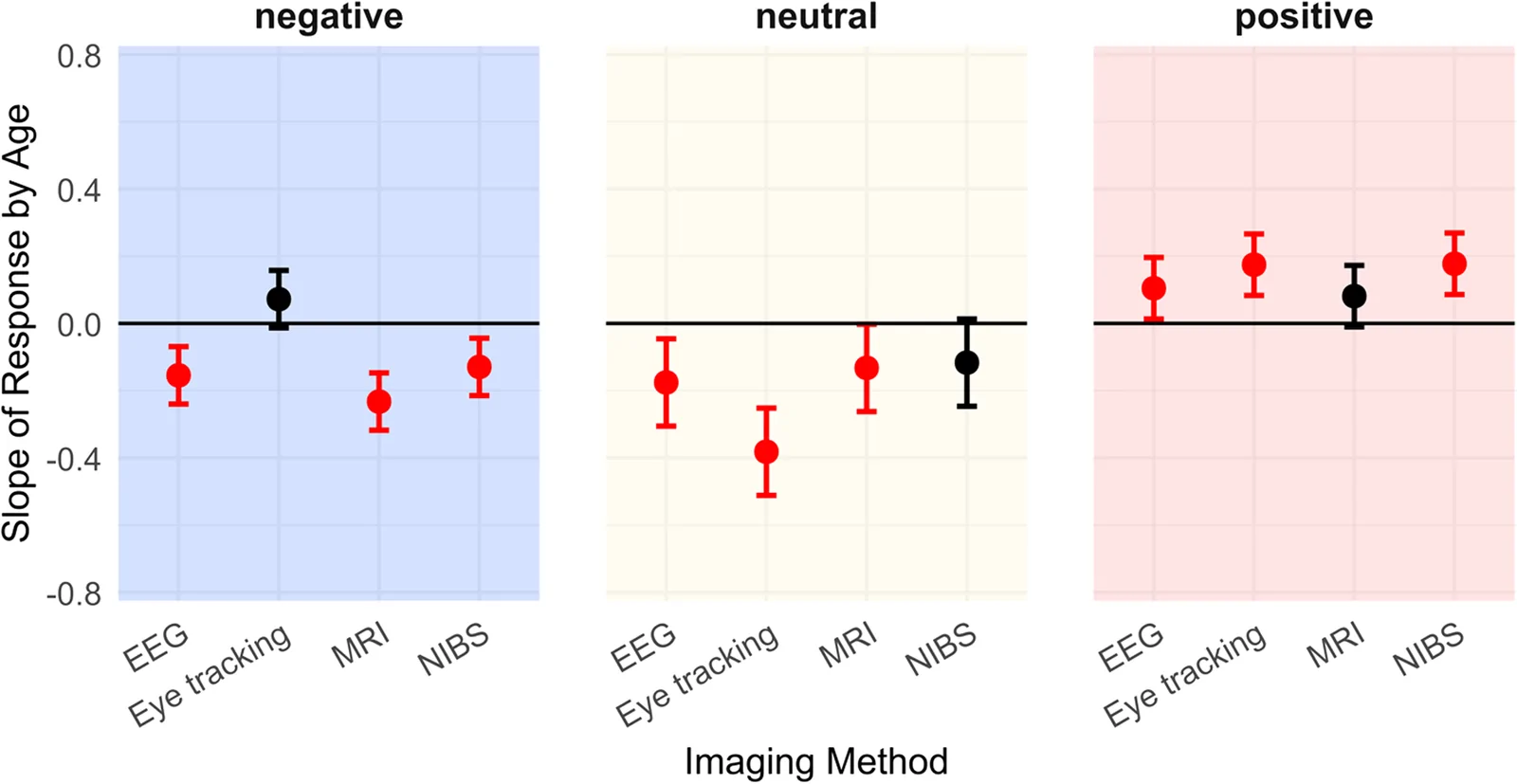
Estimated linear trends (slopes) of emotional response by age, for each of the 4 imaging methods. Error bars represent 95% confidence intervals. Points shown in red reached *p* < 0.05 and in black where *p* > 0.05. MRI = magnetic resonance imaging, EEG = electroencephalography, NIBS = non-invasive brain stimulation

We then focused on the 3-way interaction between *age*, *imaging method* and *emotion* [F(6, 21,122) = 8.07, *p* < 0.0001] to assess whether there were age-related differences in the ratings of emotions towards the four imaging methods (Fig. 3). Although several age-by-emotion slopes reached statistical significance, their magnitudes were small, consistent with the relatively flat age trends observed in Fig. 1. We found that the general slope for negative emotions showed slight decreases with advancing age for EEG, MRI and NIBS, while neutral emotions showed small age-related decreases with age for EEG, MRI and eye tracking. In contrast, positive emotions exhibited small increases with age for EEG, NIBS and eye tracking.

We then asked which of the four imaging methods people would be happy to take part in for either medical or research purposes. Most participants were happy to take part in eye tracking (medical: *N* = 304, 90.5%, research: *N* = 293, 87.2%), followed by EEG (medical: *N* = 294, 87.5%, research: *N* = 260, 77.4%) and there were no differences in the number of people who wanted to take part across medical and research contexts for these two methods (Eye tracking: χ^2^ = 0.2, *p* = 0.7; EEG: χ^2^ = 2, *p* = 0.1). For MRI (medical: *N* = 299, 89.0%, research: *N* = 234, 69.6%) and non-invasive brain stimulation (medical: *N* = 235, 69.9%, research: *N* = 154, 45.8%), more people were willing to take part in medical, rather than research studies, using these methods (MRI: χ^2^ = 8, *p* = 0.005; NIBS: χ^2^ = 17, *p* < 0.0001).

#### Motivators and barriers for participating in research studies

**Fig. 4:**
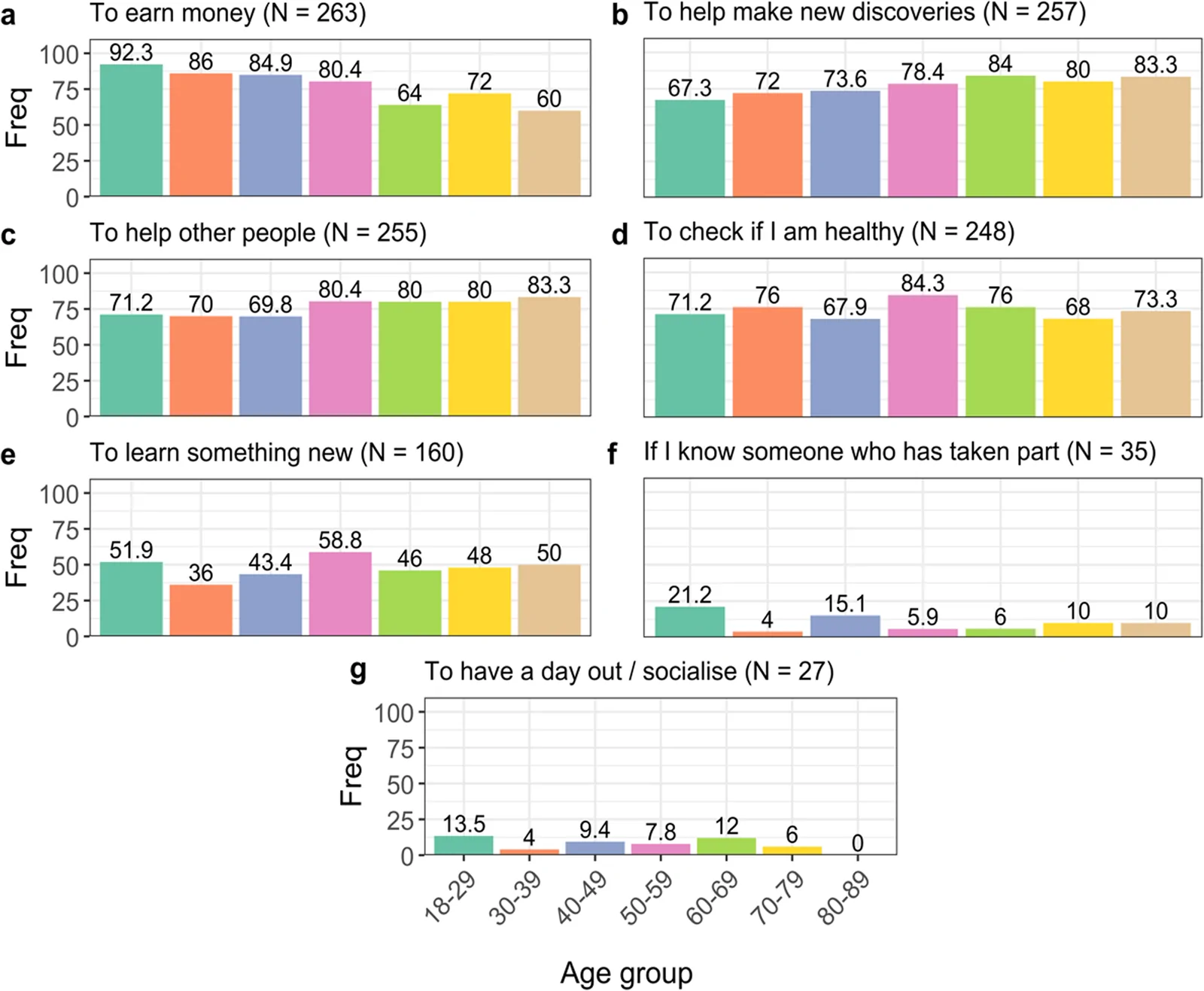
Frequency (%) of participants (*N* = 336) within each age group who selected each statement in response to the question: *“What might motivate you to take part in research studies?”*

**Fig. 5:**
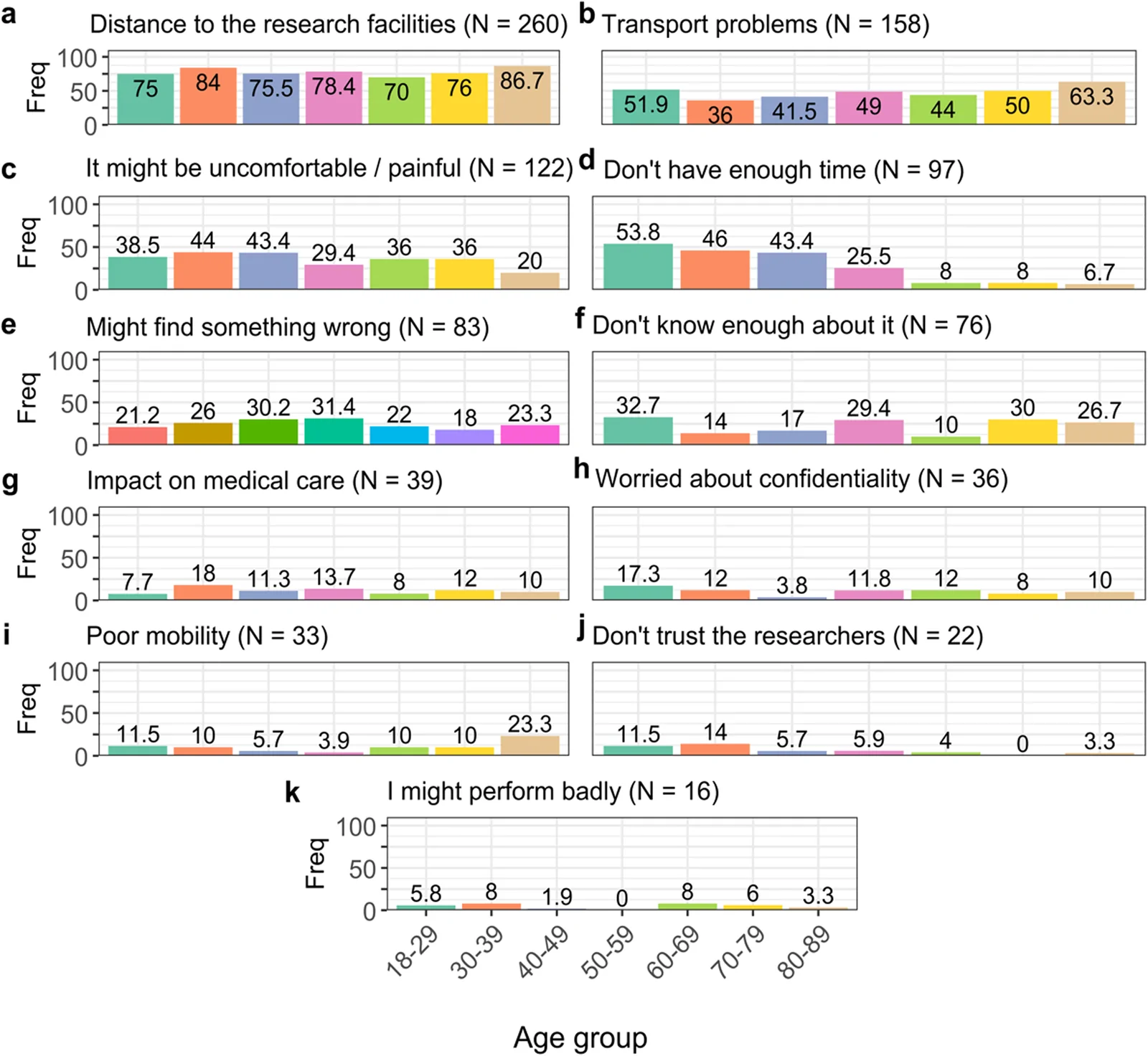
Frequency (%) of participants (*N* = 336) within each age group who selected each statement in response to the question: *“What might stop you from taking part in research studies?”*

The most frequently reported motivators were to earn money (*N* = 261, 78.3%), to help to make new scientific discoveries (76.5%, *N* = 257), to help other people (75.9%, *N* = 255), and as a health check (73.8%, *N* = 248) (Fig. 4). The most common barriers were the distance to the research facilities (*N* = 260, 77.4%), transport problems (*N* = 158, 47.0%), fear that the procedure might be uncomfortable or painful (*N* = 122, 36.3%) and lack of time (*N* = 97, 28.8%) (Fig. 5).

Younger participants were more likely to report earning money as a motivator: with every additional year of age, the odds of reporting money as a motivator decreased by 3.3% (OR = 0.97, z = −4.15, *p* < 0.001). Older participants were more likely to report helping others as a motivator: with every additional year of age, the odds of reporting helping others as a motivator increased by 1.4% (OR = 1.014, z = 1.99, *p* = 0.046). Similarly, older participants were more likely to report making new scientific discoveries as a motivator, with each additional year of age increasing the odds by 1.7% (OR = 1.017, z = 2.39, *p* = 0.017).

Age was also a factor in the barriers to taking part in research. Older participants were less likely to report concerns about the procedure being painful as a barrier: with each additional year of age, the odds decreased by 1.2% (OR = 0.99, z = −1.99, *p* = 0.047). Older participants were also less likely to report lack of time: with every additional year of age, the odds decreased by 5.3% (OR = 0.95, z = −6.71, *p* < 0.001), and were less likely to report lack of trust in researchers, with each year of age decreasing the odds by 3.9% (OR = 0.96, z = −2.86, *p* = 0.004).

Participants’ willingness to take part in research was influenced by several facilitators and barriers, after accounting for repeated measures across participants and imaging modalities.

### Facilitators

Participants were more willing to participate if they believed the study could help them check whether they were healthy or performing less well than others (Table 4). Specifically, participants endorsing this reason were over twice as likely to indicate willingness to participate across studies (OR = 2.32, *p* = 0.0075), with strong observed effects for all imaging modalities: MRI (OR = 2.5, *p* < 0.0001), EEG (OR = 3.0, *p* < 0.0001), NIBS (OR = 1.9, *p* = 0.037), and eye tracking (OR = 2.8, *p* < 0.0001). Financial incentives also had a strong effect, where participants were over three times more likely to take part for money (OR = 3.52, *p* = 0.0011), helping others increased odds by 130% (OR = 2.3, *p* = 0.031), and contributing to scientific discoveries had the largest effect, increasing odds sevenfold (OR = 7.0, *p* < 0.0001). Knowing someone who had previously participated increased willingness to participate only for NIBS studies (OR = 0.33, *p* = 0.012), suggesting that familiarity with the procedure may reduce hesitation for this modality. Age did not significantly predict participation (Table 5).

**Table 4:** Facilitators and barriers for research participation. Odds ratios (OR) and *p*-values from generalized linear mixed-effects models predicting willingness to participate across MRI, EEG, eye tracking, and NIBS studies. OR > 1 indicates increased likelihood and OR < 1 indicates decreased likelihood. Models included age and responses to facilitator and barrier items, with random intercepts for participant ID and imaging modality

Predictor	OR (95% CI)	p
Facilitators		
*Intercept*	*.11 (.018* –*.63)*	*.013*
Age	1.0 (.99–1.02)	.84
Check if I am healthy or performing less well than other people	2.32 (1.25–4.29)	.0075
Earn money	3.52 (1.66–7.48)	.0011
Help other people who might benefit from the research outcomes	2.3 (1.08–4.91)	.031
Help the researchers make new scientific discoveries	7.0 (3.17–15.45)	<.0001
Learn something about the topic	1.3 (.67–2.51)	.44
Someone I know has already taken part	.33 (.14 –.78)	.012
Have a day out/socialise	2.23 (.73–6.88)	.16
Barriers		
*Intercept*	*5.13 (.87–30.16)*	*.07*
Age	1.0 (.98–1.02)	.86
Distance to the facilities	2.68 (1.29–5.57)	.0081
Transport problems	1.19 (.63–2.26)	.59
I'm worried that it might be uncomfortable or painful	.32 (.17—.61)	.0005
I don't have enough time	1.14 (.56–2.33)	.71
I'm worried they might find something wrong with me	.49 (.25—.99)	.046
I don't know enough about it	.31 (.15—.66)	.0022
I'm worried it might impact my medical care	.84 (.31–2.26)	.73
I'm worried about confidentiality or privacy	.64 (.23–1.81)	.40
Poor mobility	1.57 (.53–4.69)	.42
I don't trust the researchers	2.07 (.56–7.58)	.27
I'm worried about performing badly	.33 (.08–1.33)	.12

**Table 5:** Random intercepts for imaging modalities. Estimated random intercepts from generalized linear mixed-effects models for facilitators and barriers. Positive values indicate that participants were more likely to report willingness (facilitators) or be less deterred (barriers) for that imaging modality relative to the overall average; negative values indicate lower likelihood. Models included random intercepts for participant ID

Imaging modality	Facilitators (intercept)	Barriers (intercept)
Eye tracking	.22	.17
EEG	1.33	1.21
MRI	-.45	-.48
NIBS	−2.05	−2.09

### Barriers

Participants were most likely to be discouraged from taking part by procedures perceived as uncomfortable or painful. For example, participants were 60% less likely to participate in MRI studies if they expected discomfort (OR = 0.40, *p* < 0.001), 48% less likely for EEG studies (OR = 0.52, *p* = 0.029), and 44% less likely for NIBS studies (OR = 0.56, *p* = 0.022). Eye tracking discomfort had a nonsignificant effect (OR = 0.65, *p* = 0.25). Distance to research facilities was another substantial barrier: participants were more than twice as likely to decline participation in MRI (OR = 2.41, *p* = 0.0038) and EEG (OR = 2.53, *p* = 0.0044) studies when travel was inconvenient. Lack of knowledge about the method also reduced willingness, with participants 63% less likely to participate in EEG studies (OR = 0.37, *p* = 0.0028) and 53% less likely in NIBS studies (OR = 0.47, *p* = 0.016). Other less common barriers included fear of performing poorly (eye tracking only) and concerns about medical findings, time, mobility, transport, confidentiality, or trust in researchers. Notably, age was not a significant predictor of any barriers, suggesting these factors influenced participants consistently across the adult age range.

### Random effects

The random effects for imaging modality indicated differences in baseline participation likelihood. In both models, NIBS showed the largest negative deviation from the overall intercept (− 2.05 in the facilitators model; − 2.09 in the barriers model), indicating substantially lower willingness to participate even after accounting for individual differences and reported facilitators and barriers. In contrast, eye tracking showed the strongest positive deviation (+ 1.33 and + 1.21), with MRI (− 0.45 to − 0.48) and EEG (+ 0.17 to + 0.22) showing relatively modest effects.

In the facilitators model, a positive intercept indicates greater-than-average willingness to participate in that modality, whereas in the barriers model a positive intercept indicates a lower-than-average tendency to report deterrents. Thus, the same sign can reflect different behavioural tendencies depending on whether the model captures willingness or perceived barriers.

#### How do people prefer to find out about research projects?

The majority of respondents preferred to find out about research studies either online (*N* = 281, 83.6%) or via their general practitioner (GP)/family doctor (*N* = 245, 72.9%). The least favoured option was being approached by someone on the street (*N* = 4, 1.2%). To examine age differences in recruitment preferences, regression analyses were conducted with age as a predictor of whether participants endorsed each method. Younger participants were more likely to prefer online sources (F = 6.45, *p* = 0.012) and social media (F = 38.42, *p* < 0.001) compared to older participants. Marginal effects analyses indicated that increasing age was associated with a small but reliable decrease in the likelihood of preferring online recruitment (average marginal effect = −0.26 percentage points per year, 95% CI [−0.48, −0.04], *p* = 0.018), although predicted probabilities showed that preference remained high across the age range, declining from 90% at age 25 to 77% at age 75. Age was also associated with a larger decrease in the likelihood of preferring social media recruitment (−0.75 percentage points per year, *p* < 0.001), with predicted probabilities declining from 47% at age 25 to 7% at age 75.

There were also small age differences in the people who did, and did not, want to find out about research via a radio advert (*F* = 4.62, *p* = 0.032) and via an advert in a local shop (*F* = 5.05, *p* = 0.025) but the total numbers who wished to find out this way were considered too small to be meaningful (*N* = 11 and *N* = 9 respectively).

Finally, just over half of the participants (53.6%, *N* = 180) indicated that they would be more likely to take part in research if they were able to watch a short video about what was involved before they arrived, with a further 131 (39%) responding that this may prompt them to participate. Indeed, 62.8% (*N* = 211) indicated that they would be more likely to take part if someone from the research team contacted them beforehand to explain what was involved, with another 108 (32.1%) responding “maybe”. The majority (88.4%, *N* = 297) wanted to see their own individual results at the end of the study, and 75.3% (*N* = 253) wanted to see how the researchers used the results, for example, the published journal articles. None of these four factors differed by the participant’s age (*p-*values = 0.23 to 0.97).

## Discussion

We used a mixed methods approach to understand the public understanding of, and attitudes towards, four different imaging methods that are commonly used in cognitive neuroscience research: Magnetic Resonance Imaging (MRI), Electroencephalography (EEG), Non-Invasive Brain Stimulation (NIBS) and Eye tracking. Two focus groups were conducted with 11 healthy adults aged 55–73, together with an online questionnaire completed by 336 people aged 18–88. Specifically, we aimed to compare the attitudes, motivators and barriers for taking part in research experiments between young and older adults, and identify potential age differences in preferred recruitment and data dissemination strategies.

### Importance of information and transparency

Three main themes emerged from the qualitative analysis of the two focus groups involving healthy older adults. The first theme was the need for reassurance through having sufficient information and transparency about the aims of the research, the safety of the procedures, and what would happen to them during the testing sessions. Many of the participants expressed anxiety around not knowing what would happen to them, particularly when asked to engage with unfamiliar technologies. These anxieties were echoed in the questionnaire results, where participants who identified a lack of study information as a barrier were less likely to participate in studies specifically involving EEG and non-invasive brain stimulation, and this effect was consistent across age groups. While previous research has identified concerns about loss of control following randomisation as a barrier to clinical trial recruitment [20, 34], we show here that information and transparency are not only relevant in clinical trial contexts, but also play an important role in shaping willingness to participate in non-clinical cognitive neuroscience research, particularly when methods are unfamiliar or perceived as invasive. However, the provision of study information must be carefully balanced, as excessive detail, particularly when presented in technical or unfamiliar language, can reduce both recruitment and retention rates [3, 30]. Co-producing participant-facing materials with members of the target group is likely to improve the acceptability and accessibility of the recruitment, information and consent forms [27], and could lead to improved experience of the research process and increased participation rates.

Developing stronger relationships between researchers, participants, and the wider public could also prove beneficial for improving understanding and transparency around research. Access to information was the main difference to emerge between the urban and rural dwelling focus groups, with the rural group only having a single point of contact (the present research team) and the urban group having multiple opportunities to participate in research studies within the local area. This suggests that geographical context may shape how individuals come into contact with research opportunities, with rural participants having fewer places where research opportunities are advertised or discussed. Such disparities may contribute to uneven patterns of engagement in neuroimaging research, particularly when participation requires travel to specialist centres typically located in urban hubs. These urban–rural differences also have implications for equal access to research. Participants living in rural areas often face greater logistical barriers, including travel distance, limited public transport, and fewer local research facilities. These constraints may restrict opportunities for participation and contribute to under-representation of rural populations in cognitive neuroscience. From the questionnaire data, younger people were more likely than older adults to report lack of trust in the researchers as a barrier to participation in research, although reassuringly this concern was relatively uncommon overall (reported by 11.5% of 18–29 year olds compared to just 3.3% of those aged 80–89). This could be achieved through public engagement and outreach activities, offering hands-on demonstrations of imaging methods, explanations of how research data is used, and opportunities for direct discussion between researchers and the public (see our previous example of this in Turner et al. [43]).

Notably, younger participants were more concerned than older people about research procedures being painful. Overall, just over one-third of participants identified potential pain as a barrier to participation, however this concern could likely be mitigated through clearer and more proactive communication during the recruitment process. Indeed, over half of respondents indicated that watching a short video explaining the procedures would promote recruitment, and two-thirds reported increased willingness to take part if they were contacted directly by the research team to discuss the study. Specifically in relation to the four imaging modalities, knowing someone else who had taken part in a NIBS study was an incentive for participation, again indicating that having information and being able to discuss the procedure is beneficial to recruitment.

Generally, positive emotions towards EEG, NIBS and eye tracking methods increased slightly with age, while negative emotions towards EEG, MRI and NIBS showed small but significant decreases. This overall shift towards more positive and less negative emotional responses may reflect increased familiarity in older adults, since eye tracking is similar to the tests experienced during routine optical examinations. It may also be explained by the “positivity effect” [10], where compared to younger people, older adults preferentially attend to positive rather than negative information, which may be related to age-related goal and perspective shifts in older age [28, 33]. This could explain why many older participants highlighted the social and interpersonal value of taking part, as these aspects may feel particularly meaningful to them. Importantly, although these age-related slopes are statistically significant, their magnitudes are modest, indicating subtle rather than pronounced changes in emotional responses across adulthood.

### Distinction between medical and non-medical research

The second theme to emerge from the older adult focus groups was the distinction that participants made between medical and non-medical research. They felt that it was important to know from the outset whether the research was aimed at developing new medical treatments, or at understanding brain mechanisms at a theoretical level. Given the emphasis of the older adults on the “collective good”, it could be that basic science research was viewed as less immediately beneficial to individuals or society, even if its importance for longer-term medical advancements was acknowledged. This indicates that the way in which researchers frame basic science studies can influence participation interest. For example, highlighting how these mechanistic studies can inform future treatments and diagnostics could bridge the gap between theoretical science and its wider benefits, particularly when the research is not explicitly framed within a medical context.

Participants also expressed a desire to use research tests as a proxy health check, which could explain the importance of knowing the aim of the study. Medical and epidemiological research, in particular, may attract individuals who are seeking health monitoring [2]. Consistent with this, our questionnaire results showed that the strongest motivator for taking part in all four imaging modalities was whether the tests could determine whether they were healthy, or performing less well than other people. This finding is consistent with a survey of participants in a cardiovascular disease prevention clinical trial which, like our two focus groups, also recruited participants across Scotland, and which found that ongoing health monitoring was the primary motivator for recruitment and retention [41]. Healthy older adults in the UK tend not to receive routine cognitive assessments unless they present to medical professionals with symptoms of decline, so participation in research may be their only opportunity to access such reassurance. It could also be that the people who answered our questionnaire may be a self-selecting cohort of people who are generally more concerned about their health and who are motivated to take part in other types of research.

The questionnaire results indicated that the prospect of undergoing imaging as part of a medical care package evoked stronger negative emotional responses compared to undergoing the same procedures within a pure research context. This is perhaps unsurprising, since medical imaging scans are typically associated with diagnostic procedures to investigate suspected illness, which would naturally evoke stronger feelings of worry and anxiety. Although we did not expand on the reasons for this in detail, our results suggest that the context and perceived purpose of imaging does shape participant attitudes towards the research project. Related to this, the focus group participants explained that having a personal connection to the research topic also influenced willingness to participate. For example, they reported being more likely to volunteer for dementia-related studies if they knew someone who had been diagnosed with dementia, indicating that familiarity with the condition may make the research feel a more personally meaningful and worthwhile time investment [39].

### Altruism and the collective good

The older adults in the focus group talked about taking part in research as an inherently altruistic act, and something proactive that they could do to contribute to the collective good. Again, this aligned with the results of the questionnaire which found that altruism towards other people who might benefit from the research outcomes was predictive of willingness to participate in all four imaging modalities, and motivation to help researchers make new scientific discoveries was associated with increased willingness to take part in MRI, EEG and NIBS, but not eye tracking. McCann et al., [29] coined the term “conditional altruism” to describe an initial willingness to take part in clinical trials for altruistic reasons, but only when participants also perceive there to be either a direct personal benefit, or at least a lack of harm or discomfort, in enrolling. In clinical contexts, this often relates to expectations of improved treatments or patient-centred outcomes, whereas in non-clinical imaging research the “conditions” tend to centre on practical considerations such as time, burden and perceived discomfort rather than anticipated health benefits. Our findings here extend this concept beyond clinical trial contexts, demonstrating that conditional altruism also shapes willingness to participate in non-clinical imaging research, and that altruism varies across age groups and for studies involving different imaging modalities.

There were some key age differences in altruism as a motivator. Whereas older people reported stronger motivators of wanting to help make new scientific discoveries and helping other people, a primary motivator for younger people was earning money. This is probably at least partially reflective of younger people being typically financially less stable than older people, although 60% of people in the oldest age bracket 80–89 years old still reported that earning money would be an incentive (vs 92.3% in the youngest group) which may reflect our recruitment via the Prolific platform. However, a recent meta-analysis of altruistic tendencies across the lifespan identified an age-related increase in altruism that was not moderated by income, sex or educational level [40]. The overall increase of positive emotions with age, alongside the reduction in negative emotions for EEG, MRI, and NIBS, may be interpreted through Socioemotional Selectivity Theory [10], which proposes that as people age, they selectively attend to more positive information over negative, and prioritising emotionally meaningful experiences.

Finally, negative emotions were more pronounced in medical compared with research contexts. This is consistent with previous work showing that anticipatory anxiety about the medical procedure itself, including fear related to the test, acts as a barrier to participation in health screening and related medical activities and highlights the importance of context and perceived risk in shaping emotional responses [1, 14, 15]. Together, these multidimensional mechanisms provide a deeper theoretical understanding of the age-related patterns observed in both altruistic motivation and emotional responses to research participation.

### Strengths and limitations

Our study had several strengths. Firstly, the focus groups included older participants from urban and rural areas, with good and poor access respectively to local research facilities, located in either universities or hospitals. The interview questions for the focus group were co-developed with a local Patient and Public Involvement group, to identify the most relevant questions to promote discussion around these themes. We also had a wide age range in the questionnaire arm, which allowed us to look at attitudes, barriers and motivation to participation across the lifespan.

In terms of limitations, all our questionnaire participants were recruited online, via the Prolific recruitment platform, and thus we have no comparator group to understand participants who may not have purposely signed up to take part in online research for money, or who may be less comfortable engaging with technology. This may have inflated the number of older people who wanted to use online methods to find out about research studies. Similarly, although we aimed to identify factors that may widen participation in research, our sample was not demographically diverse: 94.4% identified as White, 67.6% as female, and nearly one third had a bachelor’s level education. As a result, the conclusions of this study are most directly applicable to White, relatively highly educated individuals. Future studies should incorporate greater representation of ethnic minorities and individuals with lower educational attainment, for example through stratified sampling approaches, to improve generalisability. We also did not record indicators of socioeconomic status or deprivation, such as income, and almost 80% of participants were already research-active, having taken part in a research study previously. Additionally, although our focus group participants viewed short videos showing the imaging modalities, the questionnaire participants were only provided with a brief description of each of the methods, accompanied by a single static image. This is less information than participants would normally receive in a standard information sheet if they were being recruited to take part in a real cognitive neuroscience experiment and may have limited their understanding of what each method entailed and increased wariness of the procedures.

To address these limitations, future studies could examine how the format and depth of information presentation (e.g., text, images, videos) influence emotional responses and willingness to participate, to ensure comparability across imaging methods and minimise potential biases arising from differing levels of pre-existing familiarity. Researchers could also adopt community cooperative recruitment strategies, for example by collaborating with general practitioners or community centres in rural areas to improve regional sampling diversity. In addition, to reduce cognitive bias and ensure consistency in study information delivery, a multi-modal approach combining short videos with written text could be used to communicate study procedures clearly and accessibly. Implementing these strategies may enhance participation and the generalisability of findings, particularly in older adult populations.

Several researchers have produced detailed recommendations for promoting good practice when recruiting older people into medical, health and social care research [14, 15]. Here, we present the following recommendations to complement these best practice guidelines, with a particular focus on the recruitment of healthy older adults into cognitive neuroscience experiments:

**Table:** Actively supporting the inclusion of older adults in research requires designing studies that are accessible, relevant and inclusive from the outset. The following recommendations outline practical steps that research teams can take to achieve this

1. **Use targeted recruitment materials.** Information sheets, consent forms, and other documentation should be clear and accessible, and presented in plain language with minimal jargon. There should be transparency about the aims of the research. Ideally these should be co-produced with older people from diverse socioeconomic backgrounds and geographical locations
2.** Provide information through multiple channels.** In addition to written materials, online information resources and the use of videos that clearly show and explain study procedures, potential discomforts and risks may reduce anxiety and improve understanding of the study demands. Opportunities for question and answer sessions, telephone discussions, testimonials from other older participants, and open days at research centres may build trust and clarify expectations. Providing “mock scanner” setups to simulate the imaging experience prior to testing can also reduce anxiety, particularly for those who may be apprehensive about enclosed spaces or unfamiliar procedures, or physical challenges such as getting on and off the scanner table or remaining still during scanning
3. **Develop and maintain researcher-participant relationships.** Building sustained relationships can improve both recruitment and retention. Developing a dedicated older adult participant pool, in which potential participants are regularly informed about new study opportunities, provides continuity and a single point of contact for information. Involving older people in co-producing study designs and materials also promotes agency and a sense of ownership over the research process
4. **Leverage mobile technologies when appropriate.** Recent developments in mobile imaging technologies (e.g., mobile EEG and eye tracking) mean that some datasets can be collected outside of the lab-based environment and in settings that are more comfortable and familiar to participants. This could improve access to imaging research by minimising logistical barriers such as poor access to transport, limited mobility, and time constraints
5. **Engage the wider public.** Outreach activities, such as talks, workshops, open days, and information stalls, can provide an accessible means to disseminate information about cognitive neuroimaging methods and participation opportunities. These events can help build understanding and trust between researchers and the public, and provide a relaxed platform for informal questions and conversations. Public engagement can also play a valuable role in reaching a more diverse and representative sample of participants, who may feel encouraged to take part in future research studies
6. **Close the loop.** Feeding the results of the study back to participants to show how their data has been used can improve trust between researchers and participants [25, 31]. This can be in the form of overall lay summaries and/or individual results and should be provided in different formats e.g. website and physical newsletters. Keeping this feedback loop open over time allows participants to remain informed about the evolving impact of their contributions, reinforcing the sense of purpose and engagement that motivates participation in the first place. Research teams should consider building infrastructure or partnerships (e.g., with community groups or patient networks) that can sustain this cycle of information-sharing beyond the initial study period

In conclusion, the results from this mixed-methods study show that adults of all ages express a desire to be involved and included in cognitive neuroscience research, but the approaches researchers use to recruit participants may need to be tailored across both age groups and imaging methods. By integrating qualitative focus group data with a large questionnaire dataset, we provide here a richer understanding of the factors that facilitate and prevent participation. These findings point to the value of clear explanations about study procedures, a consideration of what drives participants to engage, and offers practical guidance for designing recruitment approaches that are inclusive and effective across the adult lifespan.

## Supplementary Information


Additional file 1.


## Data Availability

The data for the quantitative arm of the study is available at https://osf.io/y9fjk/. De-identified transcripts from the focus groups can be obtained on request from the corresponding author.
