# Supplementary material for: Widening participation in cognitive neuroscience: a mixed-methods study of age-related motivators, barriers and attitudes towards imaging methods
Source: BMC Med Res Methodol. 2026 Jun 19;26:192. doi: 10.1186/s12874-026-02911-3 (PMC13523283; doi:10.1186/s12874-026-02911-3)
Supplement: Supplementary file 1 [file 12874_2026_2911_MOESM1_ESM.pdf]

Modality	Setting	Affect	Alpha
MRI	Medical	Positive	0.662
MRI	Medical	Negative	0.950
MRI	Research	Positive	0.758
MRI	Research	Negative	0.954
EEG	Medical	Positive	0.658
EEG	Medical	Negative	0.965
EEG	Research	Positive	0.761
EEG	Research	Negative	0.969
NIBS	Medical	Positive	0.741
NIBS	Medical	Negative	0.966
NIBS	Research	Positive	0.831
NIBS	Research	Negative	0.970
Eye tracking	Medical	Positive	0.749
Eye tracking	Medical	Negative	0.955
Eye tracking	Research	Positive	0.777
Eye tracking	Research	Negative	0.967

Table S1. Internal consistency (Cronbach's alpha values) for positive and negative emotion scales across imaging modalities and settings.
